# Maternal Uterine Artery Adenoviral Vascular Endothelial Growth Factor (Ad.VEGF-A_165_) Gene Therapy Normalises Fetal Brain Growth and Microglial Activation in Nutrient Restricted Pregnant Guinea Pigs

**DOI:** 10.1007/s43032-024-01604-w

**Published:** 2024-06-21

**Authors:** M. D. Hristova, T. Krishnan, C. A. Rossi, J. Nouza, A. White, D. M. Peebles, N. J. Sebire, I. C. Zachary, A. L. David, O. R. Vaughan

**Affiliations:** 1 Elizabeth Garrett Anderson Institute for Women’s Health, 86-96 Chenies Mews, University College London, London, WC1E 6HX UK; 2 Biological Services Unit, Royal Veterinary College, London, UK; 3 Great Ormond Street Institute of Child Health, University College London, London, UK; 4 Centre for Cardiovascular Biology and Medicine, Division of Medicine, University College London, London, UK

**Keywords:** Placenta, Cerebral palsy, Pre-eclampsia, Ionized calcium binding adapter molecule 1 protein, Glial fibrillary astrocytic protein

## Abstract

Fetal growth restriction (FGR) is associated with uteroplacental insufficiency, and neurodevelopmental and structural brain deficits in the infant. It is currently untreatable. We hypothesised that treating the maternal uterine artery with vascular endothelial growth factor adenoviral gene therapy (Ad.VEGF-A_165_) normalises offspring brain weight and prevents brain injury in a guinea pig model of FGR. Pregnant guinea pigs were fed a restricted diet before and after conception and received Ad.VEGF-A_165_ (1 × 10^10^ viral particles, *n* = 18) or vehicle (*n* = 18), delivered to the external surface of the uterine arteries, in mid-pregnancy. Pregnant, ad libitum-fed controls received vehicle only (*n* = 10). Offspring brain weight and histological indices of brain injury were assessed at term and 5-months postnatally. At term, maternal nutrient restriction reduced fetal brain weight and increased microglial ramification in all brain regions but did not alter indices of cell death, astrogliosis or myelination. Ad.VEGF-A_165_ increased brain weight and reduced microglial ramification in fetuses of nutrient restricted dams. In adult offspring, maternal nutrient restriction did not alter brain weight or markers of brain injury, whilst Ad.VEGF-A_165_ increased microglial ramification and astrogliosis in the hippocampus and thalamus, respectively. Ad.VEGF-A_165_ did not affect cell death or myelination in the fetal or offspring brain. Ad.VEGF-A_165_ normalises brain growth and markers of brain injury in guinea pig fetuses exposed to maternal nutrient restriction and may be a potential intervention to improve childhood neurodevelopmental outcomes in pregnancies complicated by FGR.

## Introduction

Fetal growth restriction (FGR) is an obstetric complication defined by the failure of a fetus to reach its full intrauterine growth potential. It affects approximately eight percent of all viable pregnancies [1]. Of those fetuses affected by FGR, about one in five-hundred cases will be defined as both severe and early onset, occurring prior to 32 weeks gestation and leading to growth less than the third centile [2]. There is currently no treatment for FGR, even though it causes substantial perinatal morbidity and mortality [3].

FGR is a major risk factor for cerebral palsy, irrespective of whether the infant is born at or before term, or whether they experience birth asphyxia [4]. FGR is also strongly associated with poor neurodevelopmental and cognitive outcomes in childhood [5–8]. Compared to appropriately grown infants, the ones affected by FGR have less mature behavioural and neurodevelopmental scores, which are associated with lower brain volumes and structural impairments persisting at least until school-age [9–12]. Similarly, studies that induce FGR in experimental animals using nutritional, environmental and surgical manipulations demonstrate altered brain structure and behaviour in the offspring [13–19].

We previously demonstrated that uterine artery application of an adenoviral vector expressing vascular endothelial growth factor-A_165_ (Ad.VEGF-A_165_) augments uterine blood flow and mitigates FGR in pregnant sheep [20–23]. Ad.VEGF-A_165_ also increases fetal weight at term, without compromising offspring cardiometabolic phenotype, in nutrient restricted pregnant guinea pigs [24, 25]. Here, we studied the effects of maternal uterine artery Ad.VEGF-A_165_ gene-therapy on brain growth and tissue architecture. We hypothesised that maternal uterine artery Ad.VEGF-A_165_ gene transfer normalises brain weight and prevents brain injury in fetuses of nutrient restricted pregnant guinea pigs, which have extensive phenotypic similarities to women with pregnancies complicated by FGR [13, 26–30].

## Materials and Methods

### Animals

All procedures were conducted in accordance with the Animals (Scientific Procedures) Act 1986, under UK government Home Office personal and project licences (70/7408). The study used a total of 46 virgin female Dunkin Hartley guinea pig sows (> 700g), which were also part of previously reported studies [24, 25, 31]. Animals were singly housed under standard 12h:12h dark:light conditions, with ad libitum access to water supplemented with vitamin C throughout. A subset of sows (*n* = 36) was randomly allocated to maternal nutrient restriction (MNR) shown previously to induce phenotypic characteristics of FGR [13, 26–30]. Nutrient restricted sows were provided with 70% of normal daily food intake for ≥ 4 weeks prior to timed mating with a stud male guinea pig. Pregnancy was confirmed by ultrasonography ~ 21 days post-conception. MNR continued at 70% normal intake until mid-gestation (35 days post-conception), when it was increased to 90% of normal intake until term (60–65 days). Control sows were fed ad libitum throughout the study.

### Surgical Procedures

At mid-gestation, MNR sows were further randomly allocated to receive either adenoviral VEGF-A_165_ gene therapy targeted to the uterine arteries (Ad.VEGF-A_165_, 1 × 10^10^ viral particles suspended in pluronic gel, 1ml, *n* = 18), or vehicle (1ml pluronic gel, *n* = 18), as described [23, 24, 32]. All control fed sows received vehicle only. Briefly, sows were anaesthetised (44 mg/kg ketamine and 5 mg/kg xylazine intramuscularly, maintained by 1.5–2.0% isoflurane inhalation) and a laparotomy was performed. The uterine and radial arteries of both uterine horns were exposed and Ad.VEGF-A_165_ suspended in pluronic gel, or pluronic gel only, were applied to the outside of the vessels under direct vision. The incision was then closed with three layers of sutures, to the peritoneum, rectus sheath and skin, and the animal was recovered in a warm, quiet environment. Animals were provided with analgesia on the day of the surgery (carprofen, 4mg/kg s.c. and buprenorphine 0.05 mg/kg i.m.) and for three days thereafter (carprofen 4 mg/kg s.c. daily). The experiment therefore produced three groups of pregnant sows: untreated controls, MNR and MNR + Ad.VEGF-A_165_. Investigators conducting outcome analyses were blinded to experimental group.

A subset of sows (*n* = 31) were humanely killed near term (~ 60 days post-conception) with an overdose of anaesthetic (pentobarbitone sodium 200mg/kg i.v.). The maternal abdomen was opened and fetuses similarly killed then dissected, towel-dried and weighed. The skull of each fetus was opened and the brain removed, weighed, hemisected through the corpus callosum, then fixed in 4% paraformaldehyde overnight. Brains were subsequently dehydrated in 30% sucrose solution, frozen on dry ice then stored at -80°C. Litter means were calculated for fetal biometry measurements, such that the experimental unit is the sow. Remaining sows (*n* = 15) were allowed to deliver naturally at term and nurse their own pups (*n* = 36 in total) until weaning, 4 weeks after birth, when they were killed. Pups were then housed in same-sex groups and fed ad libitum. Five months after birth, adult offspring were killed (pentobarbitone sodium 200mg/kg i.v.) and the brain was dissected, weighed and fixed, as above. Offspring were treated as individuals in postnatal studies, therefore the study used 36 pups (Control: *n* = 10 pups from 4 litters; MNR: *n* = 13 pups from 5 litters; MNR + Ad.VEGF-A_165_: *n* = 13 pups from 6 litters).

### Histological and Biochemical Analyses

#### Tissue Processing

Histological analysis was performed on the brains of a subset of fetuses (Con: *n* = 11 pups from 3 litters; MNR: *n* = 7 pups from 2 litters; MNR + Ad.VEGF-A_165_: *n* = 7 pups from 2 litters) and adult offspring (Con: *n* = 10 pups from 4 litters; MNR: *n* = 5 pups from 4 litters; MNR + Ad.VEGF-A_165_: *n* = 10 pups from 5 litters), with pups treated as individuals in both cases. Brains were sectioned at 40µm, in the coronal plane, using a cryostat. Sixty sections were collected from each animal, in groups of 10 consecutive sections with a 400μm gap after every 10th section. Sections were placed directly onto glass slides, rehydrated at room temperature then stained according to the process to be studied.

#### Terminal Deoxynucleotidyl Transferase dUTP Nick End Labelling (TUNEL)

TUNEL was used to assess cell death in brain sections. Rehydrated sections underwent additional fixation in 4% paraformaldehyde and blocking in hydrogen peroxide:methanol (1:10, 15 min). After washing, TUNEL solution (1 μl of terminal deoxynucleotidyl transferase, 1.5 μl of 2'-deoxyuridine 5'-triphosphate, 100 μl of cacodylate buffer and 897.5 μl of H_2_O) was applied and slides were incubated for 2 h at 37°C. Slides were then transferred to stop solution (NaCl, sodium citrate trihydrate and H_2_O), washed and incubated with avidin–biotin-conjugated horseradish peroxidase (Vector Laboratories, UK). Finally, labelling was visualised using 3,3’-diaminobenzidine and H_2_O_2_, enhanced with Co/Ni. The slides were then dehydrated with xylene and mounted.

#### Immunohistochemistry

To assess microglial and astroglial activation, and myelination, brain sections were immunostained using antibodies specific to ionized calcium-binding adapter molecule 1 (IBA1), glial fibrillary acidic protein (GFAP) and myelin basic protein (MBP), respectively. Following antigen retrieval using acetone, the sections were washed, blocked in 5% goat serum (room temperature, 30 min) then incubated overnight at 4°C with rabbit-derived primary antibodies against IBA1 (Wako, USA, 1:3000), GFAP (Dako, UK, 1:6000) or MBP (Abcam, UK, 1:1000). Subsequently, they were washed and incubated for 1 h at room temperature with a goat-derived anti-rabbit biotinylated secondary antibody (1:100), and then with avidin–biotin-conjugated horseradish peroxidase (both Vector Laboratories, UK). Finally, the antigen–antibody binding was visualised by applying 3,3’-diaminobenzidine and H_2_O_2_ solution. Slides were then washed, dehydrated in xylene and mounted.

#### Microscopic Analysis

Within each brain specimen, analyses were performed separately in the cortex, hippocampus, striatum, thalamus and external capsule. TUNEL positive cells were counted manually at 20 × magnification in three random fields per brain region, in each of six slides from every animal. For each brain region, the count within each field of view was averaged per slide and then per animal. Microgliosis was assessed in IBA1 stained sections by counting microglial processes and cell bodies in a 3 × 3 mm grid at 40 × magnification, using a similar sampling process. Microglial ramification index for each region was calculated from the ratio A/B[33]. Astrogliosis and myelination were evaluated by determination of optical luminosity values for GFAP and MBP stained sections, as previously described [34]. Using a Leica DM5500B microscope, photographs were taken in three different randomly sampled fields of view in each brain region, at 20 × magnification. ImageJ was then used to quantify the mean and standard deviation of the optical luminosity for each image [35]. The standard deviation was subtracted from the mean and the resultant difference was subtracted from the background optical luminosity reading for the glass slide [36]. Values were averaged across each brain region.

### Statistics

Statistical analysis was performed using GraphPad Prism software. Results are presented as mean ± SD. Data not conforming to a normal distribution were logarithmically transformed before analysis. To allow use of standard logarithms, a constant was added to data sets containing values equal to or less than zero. The overall effect of experimental treatment on continuous outcome variables was determined using one-way analysis of variance (ANOVA) with Tukey’s multiple comparisons post-hoc test. Discrete data (e.g. litter sizes) were assessed by non-parametric Kruskal–Wallis test. Categorical data (e.g. offspring sex) were assessed by chi-squared test. Two-way ANOVA was also used to determine the overall effect of fetal sex (P(Sex)) on body and organ weights, and its interaction with treatment (P(Interaction)), in individual fetuses and adult offspring. In all cases, statistical significance was taken at *P* < 0.05.

## Results

### Term Fetuses

In term guinea pig fetuses, brain weight, but not body or liver weight, differed across control, MNR and MNR + Ad.VEGF-A_165_ treatment groups (Table 1). The brains of untreated MNR fetuses tended to be lighter than those of control normally grown fetuses (*P* = 0.065, Tukey’s post-hoc). By contrast, brains of treated MNR fetuses from nutrient restricted sows that received Ad.VEGF-A_165_ were heavier than those of untreated MNR fetuses (*P* = 0.023) and did not differ from normally grown controls (*P* = 0.99). The overall effects of FGR and Ad.VEGF-A_165_ were similar when fetal brain weight was expressed as a percentage of body weight (Table 1). Brain weight did not differ across groups when expressed as a percentage of liver weight. Gestational age at necropsy, litter size and the proportion of fetuses of each sex was the same in all three groups (Table 1). Fetal body, brain and liver weights were not affected by fetal sex (Two-way ANOVA, *P*(Sex) > 0.05), or the interaction between fetal sex and treatment group (Two-way ANOVA, *P*(Interaction) > 0.05).

**Table 1:** Biometry of fetuses and adult offspring of control, maternal nutrient restricted (MNR) and maternal nutrient restricted pregnant guinea pigs given Ad.VEGF-A_165_ gene therapy (MNR + Ad.VEGF-A_165_)

	Control		MNR		MNR + Ad.VEGF-A165		Overall *P* value
	Mean	SD	Mean	SD	Mean	SD	
*Fetuses*	*n* = 6 litters		*n* = 13 litters		*n* = 12 litters		
Brain weight (g)	2.5 ±	0.1	2.3 ±	0.3	2.5 ±	0.2†	***0.02***
Body weight (g)	85 ±	7	85 ±	12	84 ±	11	> 0.05
Liver weight (g)	4.5 ±	0.6	4.0 ±	1.1	4.4 ±	0.9	> 0.05
Brain/body weight (%)	3.0 ±	0.2	2.7 ±	0.4	3.0 ±	0.3†	***0.03***
Brain/liver weight (%)	58 ±	8	58 ±	14	60 ±	15	> 0.05
Gestational age (days)	61 ±	1	61 ±	1	61 ±	2	> 0.05
Litter size	4.0 ±	1.5	3.0 ±	2.5	3.0 ±	1.8	> 0.05^a^
Sex (% female)	54		45		50		> 0.05^b^
*Adult offspring*	*n* = 10		*n* = 13		*n* = 13		
Brain weight (g)	3.9 ±	0.4	3.8 ±	0.3	3.7 ±	0.4	> 0.05
Body weight (g)	880 ±	122	787 ±	88	847 ±	119	> 0.05
Liver weight (g)	27 ±	5	26 ±	4	26 ±	4	> 0.05
Brain/body weight (%)	0.45 ±	0.07	0.48 ±	0.07	0.43 ±	0.07	> 0.05
Brain/liver weight (%)	15 ±	3	15 ±	3	15 ±	3	> 0.05
Postnatal age (days)	167 ±	13	147 ±	27	142 ±	31	> 0.05
Litter size	2.5 ±	2.5	4.0 ±	1.0	3.5 ±	2.3	> 0.05^a^
Sex (% female)	60		62		54		> 0.05^b^

Overall effect of maternal treatment determined by one-way ANOVA, unless indicated ^a^ Kruskal–Wallis test or ^b^ Chi-square test. Post-hoc comparisons between groups performed by Tukey multiple comparisons test, with *P < 0.05 versus control and †*P* < 0.05 versus FGR. Values are mean ± SD

**Fig. 1:**
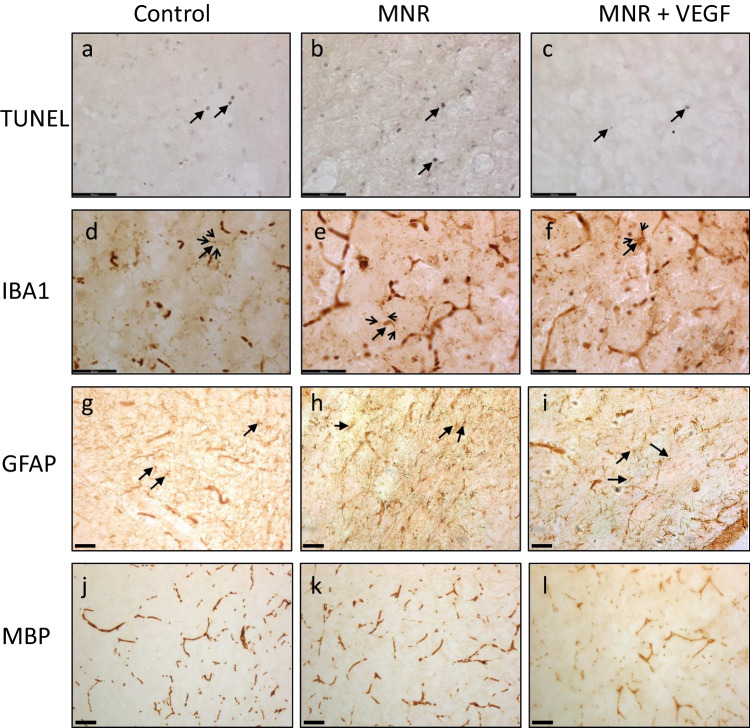
Representative images of histological analyses in brains of fetuses of control, maternal nutrient restricted (MNR) and maternal nutrient restricted pregnant guinea pigs given Ad.VEGF-A_165_ gene therapy (MNR + Ad.VEGF-A_165_). (**a-c**) TUNEL stained images of cerebral cortex, 40 × magnification, apoptotic nuclei indicated (closed arrow); (**d**-**f**) cortex immunostained for IBA1; 40 × magnification, microglial cell bodies (closed arrow) and processes (open arrow) indicated; (**g**-**i**) external capsule immunostained for GFAP, 20 × magnification, astroglial cells indicated (closed arrow); (**j**-**l**) thalamus immunostained for MBP, 20 × magnification. Scale bar 59.5µm

Microglial ramification, assessed through IBA1 immunoreactivity of brain sections, was greater in MNR compared to control fetuses in all anatomical regions of the brain that were studied (Table 2, Fig. 1d-f). However, microglial ramification in the hippocampus, striatum and thalamus of treated MNR fetuses from nutrient restricted dams that received Ad.VEGF-A_165_ was lower than that in untreated MNR fetuses and similar to control values. Ramification also tended to be lower in the cortex and external capsule of treated MNR + Ad.VEGF-A_165_ fetuses compared to untreated MNR fetuses, albeit the difference was not statistically significant (*P* = 0.06 and *P* = 0.27 respectively, Tukey’s post-hoc). Neither TUNEL positive cell count nor optical luminosity values for astrogliosis (GFAP immunoreactivity) and myelination (MBP immunoreactivity) differed between the three groups of fetuses, at term (Table 2, Fig. 1).

**Table 2:** Histological indices of brain injury in fetuses of control, maternal nutrient restricted (MNR) and maternal nutrient restricted pregnant guinea pigs given Ad.VEGF-A_165_ gene therapy (MNR + Ad.VEGF-A_165_)

		Control		MNR		MNR + Ad.VEGF-A165		Overall *P* value
		Mean	SD	Mean	SD	Mean	SD	
Fetuses		*n* = 11		*n* = 7		*n* = 7		
Cell death (TUNEL positive cells)	Cortex	2.2 ±	1.0	2.2 ±	1.1	2.5 ±	0.9	> 0.05
	Hippocampus	1.5 ±	0.7	3.2 ±	1.9	1.8 ±	0.7	> 0.05
	Striatum	1.8 ±	0.8	2.5 ±	0.9	2.3 ±	0.4	> 0.05
	Thalamus	2.5 ±	0.9	2.7 ±	1.2	3.1 ±	1.6	> 0.05
	External capsule	2.1 ±	0.6	2.3 ±	1.3	2.1 ±	1.3	> 0.05
Microgliosis (IBA1 ramification index)	Cortex	0.4 ±	0.4	1.4 ±	0.7*	0.7 ±	0.8	***0.01***
	Hippocampus	0.2 ±	0.2	1.8 ±	1.0*	0.6 ±	0.5†	***0.00***
	Striatum	0.2 ±	0.2	1.7 ±	1.2*	0.7 ±	0.6†	***0.00***
	Thalamus	0.4 ±	0.3	1.1 ±	0.7*	0.5 ±	0.5†	***0.01***
	External capsule	0.4 ±	0.4	1.4 ±	0.8*	0.9 ±	1.1	***0.07***
Astrogliosis (GFAP optical luminosity)	Cortex	47 ±	15	50 ±	12	53 ±	9	> 0.05
	Hippocampus	37 ±	14	40 ±	7	39 ±	9	> 0.05
	Striatum	29 ±	13	30 ±	5	29 ±	14	> 0.05
	Thalamus	28 ±	12	31 ±	4	33 ±	6	> 0.05
	External capsule	54 ±	18	60 ±	9	53 ±	12	> 0.05
Myelination (MBP optical luminosity)	Cortex	30 ±	9	39 ±	8	32 ±	7	> 0.05
	Hippocampus	25 ±	7	33 ±	6	31 ±	11	> 0.05
	Striatum	28 ±	9	26 ±	5	19 ±	6	> 0.05
	Thalamus	30 ±	8	35 ±	4	29 ±	4	> 0.05
	External capsule	57 ±	14	61 ±	8	54 ±	8	> 0.05

Overall effect of maternal treatment determined by one-way ANOVA. Post-hoc comparisons between groups performed by Tukey multiple comparisons test, with *P < 0.05 versus control and †*P* < 0.05 versus FGR. Values are mean ± SD

### Adult Offspring

Adult offspring guinea pigs from control, MNR and MNR + Ad.VEGF-A_165_ groups had similar brain, liver and body weights when they were studied at 5 months of age (Table 1). Male offspring were heavier than females (Two-way ANOVA, *P*(Sex) < 0.001) but their brain and liver weights were similar (*P*(Sex) > 0.05) and there was no interacting effect of sex and treatment on offspring biometry (*P*(Interaction) > 0.05). Pups originated from similarly sized litters and had similar sex distributions.

**Fig. 2:**
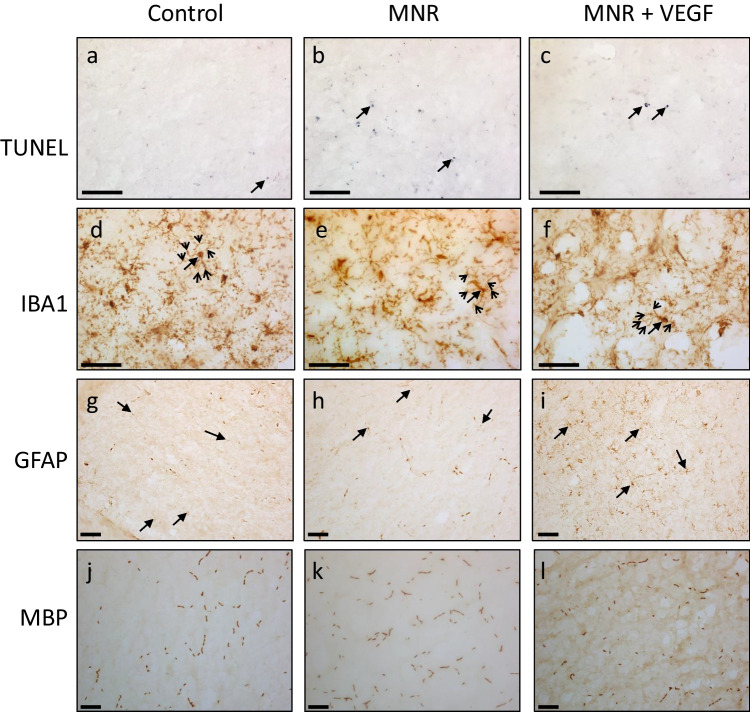
Representative images of histological analyses in brains of adult offspring of control, maternal nutrient restricted (MNR) and maternal nutrient restricted pregnant guinea pigs given Ad.VEGF-A_165_ gene therapy (MNR + Ad.VEGF-A_165_). (**a**-**c**) TUNEL stained images of external capsule, 40 × magnification, apoptotic nuclei indicated (closed arrow); (**d**-**f**) hippocampus immunostained for IBA1; 40 × magnification, microglial cell bodies (closed arrow) and processes (open arrow) indicated; (**g**-**i**) external capsule immunostained for GFAP, 20 × magnification, astroglial cells indicated (closed arrow); (**j**-**l**) hippocampus immunostained for MBP, 20 × magnification. Scale bar 59.5µm

Hippocampal microglial ramification was higher in MNR + Ad.VEGF-A_165_ adult offspring compared to either control or untreated MNR offspring (Table 3, *P* < 0.05, Tukey’s post-hoc, Fig. 2d-f). Conversely, microglial ramification in the thalamus tended to be less in MNR than control adult offspring, irrespective of whether the sow received Ad.VEGF-A_165_ (Table 3). There were no significant inter-group differences in microglial ramification in the cortex, striatum or external capsule. Astroglial activation, determined through GFAP immunoreactivity, was elevated in the external capsule in the brains of MNR + Ad.VEGF-A_165_ offspring, but not untreated FGR offspring, compared to controls (Table 3, Fig. 2g-i). Astroglial activation was not affected by treatment in any other brain region and there were no differences in TUNEL positive cell death or myelination between adult offspring in the three groups.

**Table 3:** Histological indices of brain injury in adult offspring of control, maternal nutrient restricted (MNR) and maternal nutrient restricted pregnant guinea pigs given Ad.VEGF-A_165_ gene therapy (MNR + Ad.VEGF-A_165_)

		Control		MNR		MNR + Ad.VEGF-A165		Overall *P* value
		Mean	SD	Mean	SD	Mean	SD	
Adult offspring		*n* = 10		*n* = 5		*n* = 10		
Cell death (TUNEL positive cells)	Cortex	0.0 ±	0.1	0.2 ±	0.2	0.1±	0.2	> 0.05
	Hippocampus	0.1 ±	0.1	0.3 ±	0.9	0.2 ±	0.4	> 0.05
	Striatum	0.1 ±	0.1	0.2 ±	0.3	0.0 ±	0.2	> 0.05
	Thalamus	0.1 ±	0.1	0.8 ±	1.8	0.1 ±	0.1	> 0.05
	External capsule	0.1 ±	0.1	0.6 ±	1.7	0.2 ±	0.3	> 0.05
Microgliosis (IBA1 ramification index)	Cortex	5.9 ±	1.3	7.3 ±	1.3	6.5 ±	2.4	> 0.05
	Hippocampus	4.8 ±	1.8	4.2 ±	0.7	6.7 ±	1.7*†	***0.021***
	Striatum	5.5 ±	2.9	3.5 ±	2.8	4.5 ±	4.9	> 0.05
	Thalamus	4.8 ±	1.8	2.9 ±	1.3	3.0 ±	0.6	***0.020***
	External capsule	4.5 ±	1.8	5.3 ±	2.1	5.5 ±	1.5	> 0.05
Astrogliosis (GFAP optical luminosity)	Cortex	16 ±	3	18 ±	5	22 ±	6	> 0.05
	Hippocampus	16 ±	4	14 ±	5	19 ±	5	> 0.05
	Striatum	16 ±	6	17 ±	6	21 ±	7	> 0.05
	Thalamus	14 ±	6	13 ±	3	17 ±	4	> 0.05
	External capsule	19 ±	6	21 ±	9	29 ±	7*	***0.021***
Myelination (MBP optical luminosity)	Cortex	25 ±	9	19 ±	6	25 ±	3	> 0.05
	Hippocampus	23 ±	6	17 ±	3	23 ±	3	> 0.05
	Striatum	22 ±	6	17 ±	11	21 ±	4	> 0.05
	Thalamus	29 ±	5	25 ±	7	28 ±	2	> 0.05
	External capsule	40 ±	8	36 ±	10	38 ±	3	> 0.05

Overall effect of maternal treatment determined by one-way ANOVA. Post-hoc comparisons between groups performed by Tukey multiple comparisons test, with *P < 0.05 versus control and †*P* < 0.05 versus FGR. Values are mean ± SD

## Discussion

This study shows that uterine artery gene therapy with Ad.VEGF-A_165_ in pregnant guinea pigs prevents the effects of maternal nutrient restriction on offspring brain growth and microglial activation. The results also demonstrate that brain weight and cellular composition remain normal into adulthood, in offspring of Ad.VEGF-A_165_ treated dams, without evidence of changes in cell death or myelination. Maternal Ad.VEGF-A_165_ treatment increased microgliosis and astrogliosis in some parts of the adult offspring brain but most brain regions were unaffected. Therefore, uterine artery Ad.VEGF-A_165_ gene therapy does not adversely affect offspring brain growth or cause injury at a cellular level, and may beneficially mitigate the effects of FGR in the perinatal period.

Maternal nutrient restriction reduced fetal absolute brain weight near term, consistent with previous studies in pregnant guinea pigs [13–15]. This finding also recapitulates the phenotype of human neonates affected by FGR, who have lower total brain volumes, as well as smaller gray matter and regional volumes, compared to normally grown, appropriate for gestational age infants [9, 10]. However, our finding that fetal brain weight is also reduced as a percentage of body weight contrasts with other studies showing that brain growth is relatively less affected by maternal nutrient restriction and other, more severe prenatal insults, like uterine artery ligation or maternal chronic hypoxia [13–18]. Maternal nutrient restriction in our model does not therefore appear to induce fetal brain sparing, a redistribution of blood flow to the brain at the expense of the liver and other viscera, thought to be an adaptive response to hypoxia in human fetuses with FGR [37, 38]. This discrepancy most likely relates to the mild and variable degree of fetal growth restriction produced by maternal nutrient restriction in guinea pigs, which in turn relates to varying age and body habitus at conception and was reported by us and others previously [30–32]. Indeed, birth weight was similar in control and MNR groups in the present cohort, possibly due to a tendency for lower litter size in nutrient restricted dams [27]. Mechanistically, impaired brain growth in fetuses of undernourished guinea pig dams may be explained by reduced fetal nutrient and oxygen delivery concomitant with placental vascular deficiency [13, 29].

Despite reduced brain weight in the perinatal period, offspring from nutrient restricted sows had similar sized brains to controls in adulthood. By contrast, long term studies in human neonates affected by FGR suggest that brain structural deficits persist, with reduced total and regional brain volumes reported at least until 10 years of age [11, 12, 39]. Therefore, the guinea pig brain may be more capable of postnatal catch-up growth. However, we did not investigate whether the offspring of nutrient restricted sows had persisting alterations in behaviour or cognition, which have been described both in children affected by FGR [9–11] and in other guinea pig studies of prenatal undernutrition [19].

In our study, the main effect of maternal nutrient restriction on the fetal brain at a cellular level was a reduction in microglial activation relative to fetuses of ad libitum fed control dams, as evidenced by greater ramification of IBA-1 positive cells. Microglial macrophages typically survey tissue via branching processes in the quiescent state and adopt a more rounded phagocytic morphology in the activated state, when inflammation occurs. This effect was unexpected, given that previous animal studies suggest *increased* microglial activation, with less ramification, in the brains of FGR piglets [40]. In rodents, in early perinatal life, IBA-1 positive macrophages with a rounded phagocytic morphology are mostly restricted to sub-cortical white matter. Later, as the microglial cells take up residence in the rest of the brain regions, IBA-1 positive microglial morphology becomes more ramified [41]. Therefore, greater microglial ramification in the MNR fetuses in our study could be explained by accelerated maturation or infiltration of the microglial population in the fetal brain, because we did not quantify the absolute number or density of IBA-1 positive cells present. Whatever the reason for the greater microglial ramification in the brains of fetuses exposed to maternal nutrient restriction, this effect was resolved in the adult offspring, which had similar IBA-1 ramification indices when compared to the control group. Therefore, maternal nutrient restriction did not induce lasting changes in offspring brain inflammation and glial composition (Figs. 1 and 2).

In piglets with FGR, microglial activation is accompanied by increased abundance of pro-inflammatory cytokines, reduced neuronal proliferation, astrogliosis and impaired myelination, all consistent with hypoxic brain injury [40]. These results also differ from our own, which indicate that astroglial activation, measured through GFAP immunoreactivity, and myelination, measured through MBP immunoreactivity, are similar in fetuses of nutrient restricted and control dams. In FGR guinea pigs, reduced fetal brain myelination, astrogliosis and inflammation have been reported mainly in more severe insults associated with uterine artery ligation and chronic hypoxia [16, 17, 42]. Therefore, these indices of brain injury are more likely related to marked hypoxia per se, rather than the moderate placental insufficiency induced by maternal nutrient restriction. We did not identify a difference in the number of TUNEL positive cells in the brains of MNR and control fetuses, in contrast with another study that focussed on selected FGR fetuses and found increased apoptosis in the brains of guinea pig pups exposed to similar maternal nutrient restriction [14]. In that study, apoptosis was localised to the periventricular white matter and hippocampus and evident only in fetuses below a cut-off body weight threshold of 80g. Moreover, although we did not find evidence of significant increase in TUNEL positive cells in the brains of FGR fetuses, the density was double that in ad libitum fed controls, suggesting a larger number of samples would most likely have identified a similar effect in the three-group design used in our study.

Notwithstanding the mild effects of maternal nutrient restriction on fetal brain weight and glial activation, Ad.VEGF-A_165_ normalised these effects such that fetuses of dams given gene therapy had similar brain weights and IBA-1 ramification indices to control fetuses. The data therefore suggest a beneficial therapeutic effect of Ad.VEGF-A_165_ to combat the effects of maternal nutrient restriction on the brain, in the perinatal period. Similarly, in rats, maternal antioxidant treatment with vitamin C reverses the effects of environmental hypoxia on offspring hippocampal structure, as well as improving memory function [43]. On the other hand, targeted delivery of a nanoparticle therapy designed to augment insulin growth factor 1 expression in the guinea pig placenta does not appear to alter the effect of nutrient restriction on fetal brain growth [44]. Therefore, the data suggest that prenatal interventions for FGR can indirectly protect brain development and that amelioration of uteroplacental blood flow, hypoxia and oxidative stress are more important than enhancing placental function, in this context. Certainly, maternal uterine artery Ad.VEGF-A_165_ gene therapy improves uterine artery vasodilatation in guinea pigs, in our previous studies. [20, 21, 24]. Ad.VEGF-A_165_ gene therapy also improves uterine blood flow in sheep, in association with reduced brain-sparing [22, 45].

The brains of offspring from the MNR + Ad.VEGF-A_165_ group remained similar in size to controls in adulthood, suggesting that they continued to grow normally after birth. Moreover, the increase in hippocampal IBA-1 ramification index in the adult MNR + Ad.VEGF-A_165_ group suggests a reduced inflammatory state in the brains of these animals, compared to untreated MNR offspring. This therapeutic effect could be due to Ad.VEGF-A_165_ improving uterine blood flow and reducing hypoxia and oxidative stress in the fetal brain, leading to a lasting dampening of pro-inflammatory cytokine production. The regional specificity of this long term effect of Ad.VEGF-A_165_ may be a consequence of the high metabolic rate of the hippocampus, rendering it particularly susceptible to ATP deficit [10].

Contrary to its effects on fetal brain weight and microglial activation, prenatal Ad.VEGF-A_165_ gene therapy increased GFAP positive astroglial activation in the external capsule of the adult offspring brain, indicating active repair of neuronal tissue. Although the cause of this astrogliosis is unclear, it is unlikely to be a long-term inflammatory effect of vector leak into the fetus, because our previous study shows no evidence of vector spread across the placenta in guinea pigs [24]. Older studies report that adenoviral vectors are capable of causing brain inflammation but these concerns are mitigated by the use of more modern vectors that have reached clinical trial for numerous diseases, including neurological diseases in children [46]. We cannot rule out a potential local inflammatory response, with oedema and macrophage infiltration due to overexpression of VEGF-A_165_, which we previously identified in transduced uterine and radial arteries in pregnant sheep [20]. However, using the alternative pre-processed short form VEGF-D transgene in the adenovirus (Ad.VEGF-D^ΔNΔC^) did not result in an inflammatory effect, and this is likely to be the vector of choice for clinical translation [47]. We have previously demonstrated minimal transfer of this Ad.VEGF-D^ΔNΔC^ vector across the ex vivo human placenta, making it unlikely to reach the fetal blood and have an adverse effect [48]. We also did not assess the relative abundance of pro-inflammatory A1 and protective A2 astrocytes in the present study, meaning that increased GFAP immunoreactivity was not necessarily a consequence of increased inflammation, rather than repair. Elevated astroglial activation was confined to the external capsule, with no evidence of astrogliosis in any other brain region studied. There were no accompanying alterations in apoptosis or myelination, suggesting that there are few adverse effects of maternal Ad.VEGF-A_165_ gene therapy on the offspring brain. Nevertheless, the safety of the therapy will continue to be monitored in our ongoing preclinical studies using Ad.VEGF-D^ΔNΔC^.

This study did not include animals treated with a control adenoviral vector without the VEGF-A_165_ transgene, in contrast with our previous studies, which used a β-galactosidase-expressing reporter construct in the same vector backbone (Ad.lacZ) [20–24, 49]. Our study design including nutrient-restricted and ad libitum fed controls treated with pluronic gel allowed us to assess both the efficacy and safety of the combined VEGF transgene and adenoviral vector, versus vehicle, and was most like the expected design of a randomised controlled trial in humans. The previous studies already demonstrated that Ad.VEGF-A_165_ increases activation of endothelial cell angiogenesis signalling [49], uterine artery dilatation [24], uterine blood flow [20, 21], fetal growth velocity [22] and birthweight [24] compared to Ad.lacZ. We did not consider it necessary to use more animals to include another group of nutrient-restricted, pregnant guinea pigs given Ad.lacZ in this study.

Taken together, the data support the safety and efficacy of maternal uterine artery Ad.VEGF gene therapy for fetal growth restriction. In agreement with our original hypothesis, they show that Ad.VEGF-A_165_ normalises fetal brain growth in guinea pig fetuses of nutrient restricted sows. They also indicate that Ad.VEGF-A_165_ reverses the effects of maternal nutrient restriction on microglial activation in the perinatal period. Ad.VEGF-A_165_ has few adverse effects on the brain, even when offspring are followed up in the long term. Ad.VEGF gene therapy therefore remains a promising intervention to mitigate placental insufficiency in pregnancies complicated by severe FGR. We are currently undertaking further pre-clinical studies in guinea pigs to optimise Ad.VEGF-D^ΔNΔC^ vector dosage and planning a clinical trial.

## Data Availability

The authors confirm that the data supporting the findings of the present study are available within the article and from the corresponding author [Owen Vaughan] upon reasonable request.
